# Listeria Monocytogenes Septicemia in the Setting of an Ulcerative Colitis Flare

**DOI:** 10.7759/cureus.28638

**Published:** 2022-08-31

**Authors:** Michael J Seraphin, Raul Isern, Alyssa K Maclean, Christopher M Heli, Michael B Shaw

**Affiliations:** 1 Division of Infectious Disease, Creighton University School of Medicine, Omaha, USA; 2 Department of Internal Medicine, Creighton University School of Medicine, Omaha, USA

**Keywords:** immune suppression, listeriosis, septicemia, ulcerative colitis, listeria monocytogenes

## Abstract

A 51-year-old patient with a history of ulcerative colitis was admitted after three days of bloody diarrhea and abdominal pain. The patient had been trialing different immunosuppressive therapies over the past year. An acute flare of ulcerative colitis was confirmed, and our patient began to improve upon IV methylprednisolone. Blood cultures on admission were positive for *Listeria monocytogenes*. IV ampicillin and gentamicin were begun for treatment. Upon discharge, the patient was switched to high-dose amoxicillin. This case report shows that *Listeria *can be a cause of septicemia in ulcerative colitis patients undergoing immunosuppressive therapy.

## Introduction

Ulcerative colitis (UC) is an inflammatory bowel disease characterized by inflammation of the intestinal mucosa. UC classically involves the rectum and extends proximally up the colon. The exact cause of the disease is unknown but is theorized to be due to an autoimmune inflammatory response due to colonic bacteria [1]. Treatment of UC involves immunosuppressive agents, putting patients at risk for opportunistic infections like *Listeria (L.) monocytogenes. L. monocytogenes* is a gram-positive anaerobic bacteria associated with food-borne infections in the United States [2]. Uncommonly, *L. monocytogenes* is associated with a systemic infection known as listeriosis, typically in patients with prior risk factors for disseminated infection like immunosuppression. While the exact incidence of* L. monocytogenes *infection in UC patients is unknown, previous case reports have demonstrated *L. monocytogenes* bacteremia and meningitis in UC patients in the setting of a UC flare [3].

## Case presentation

A 51-year-old male trucker presented to the emergency department (ED) at the Veterans Affairs Medical Center with a three-day history of intense abdominal pain and bloody diarrhea while on his trucking route. He had been diagnosed with UC at 19 years old and had three hospitalizations since his diagnosis. At the time of presentation, he was managing his UC with vedolizumab and was taking 35 mg prednisone (day 20 of a taper) following a recent UC flare. Previous unsuccessful medication trials in the past two years included mesalamine, adalimumab, and infliximab, all with concurrent prednisone. In the ED, the patient endorsed weakness, chills, and 8/10 abdominal pain (10/10 when straining to defecate) and denied vomiting or fever. Vitals were a temperature of 97.8℉, heart rate of 97, respiratory rate of 20, and blood pressure of 135/87. Pertinent labs included a WBC count of 20.8x10^3^/µL, procalcitonin of 0.37 ng/mL, and lactic acid of 1.7 mmol/L. CT in the ED showed diffuse mild/moderate mural thickening of the rectum and distal colon, findings consistent with an acute UC flare. Blood cultures and a BioFire FilmArray GI Panel (BioFire Diagnostics, Salt Lake City, Utah) were collected, and the patient was admitted to the hospital floor for management. Prednisone was held until the stool pathogen panel returned.

Overnight, the patient had fevers up to 102.3℉. On the second day of admission, the patient’s painful and frequent bloody diarrhea persisted. The pathogen panel returned negative, and a flexible sigmoidoscopy was performed showing diffuse colonic inflammation consistent with acute severe ulcerative pancolitis (Mayo Endoscopic Subscore of 2). Biopsies demonstrated severe active chronic colitis with ulceration and were negative for both granulomas and Cytomegalovirus (CMV) by immunostaining. At this time, 1/1 blood culture from admission returned positive for *Corynebacterium spp.* However, this report was shortly corrected to *L. monocytogenes.* Given the clinical appearance of the patient and the infrequency of *L. monocytogenes *as a blood-borne pathogen, the managing team had low suspicion for bacteremia, and repeat blood cultures were obtained. IV methylprednisolone (20 mg every 8 hours) was started to manage the UC flare. Over the next day, the patient’s stool frequency had decreased, stool consistency had improved, and his appetite returned.

**Figure 1:**
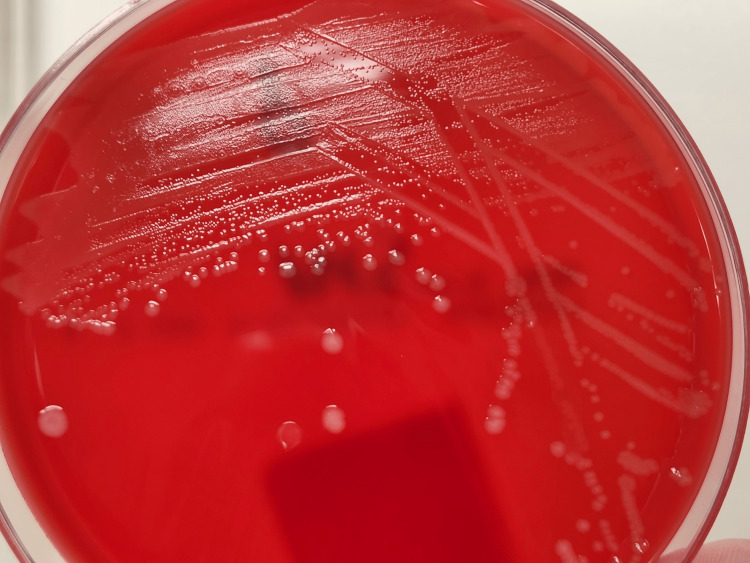
Patient's blood culture demonstrating Listeria monocytogenes narrow zones of beta-hemolysis on blood agar

On the fourth day of admission, the patient’s clinical picture was still improving, yet 2/2 of the repeat cultures were positive for *L. monocytogenes* (Figure 1). Infectious disease was consulted for a treatment plan and the patient began receiving 425 mg gentamicin every 24 hours and 2 gm ampicillin every four hours. Serial blood cultures were attained at this point to monitor response to therapy. While there was an ongoing outbreak of *L. monocytogenes* in the United States, the medical team was unable to connect the patient to any possible exposures. However, bacterial samples were sent for whole genome sequencing to determine if the pathogen was related. The patient remained well-appearing with improved stool frequency and consistency, despite continual positive blood cultures. An echocardiogram was obtained to rule out bacterial seeding of the valves and revealed no masses or vegetation. By the seventh day of admission, blood cultures had remained negative for three days. The patient was switched to high-dose amoxicillin (1000 mg every 8 hours) for 14 days in anticipation of his flight back home. He was discharged and followed closely by his primary care provider and gastroenterologist.

## Discussion

*L. monocytogenes*, while being one of the most common foodborne infections, rarely causes systemic infection. In immunocompetent patients, *L. monocytogenes* infection is usually localized gastroenteritis. The highest risk for severe infection includes patients who are immunosuppressed, pregnant, neonates, or elderly [4,5]. UC patients can potentially be at a higher risk for listeriosis due to several factors. As with our patient, the treatment for severe UC involved immune-modulating therapies, lowering the body’s ability to fight off infection [6]. Cases of *L. monocytogenes *infection have been previously noted in patients on anti-TNF treatment [7].

Another recent study demonstrated opportunistic *L. monocytogenes *meningitis infection after starting ustekinumab therapy for plaque psoriasis, another autoimmune condition. Ustekinumab is an engineered human monoclonal antibody that is targeted to block the function of interleukin-12 (IL-12) and interleukin-23 (IL-23). IL-12 and IL-23 bolster the immune response to bacterial infection by promoting macrophage ability to envelope and destroy pathogens [8]. Our patient had started ustekinumab 520 mg IV six days before presenting to the emergency department for his UC flare. We believe this medication, along with his history of other immunosuppressive medications, may have contributed to his increased risk of developing *L. monocytogenes *septicemia, given suboptimal macrophage functioning in preventing bacterial penetration through the weakened gut mucosa. 

*L. monocytogenes *can also lead to increased severity of UC flare due to increased inflammation in the colonic mucosa [3]. For patients with invasive listeriosis, complications include sepsis, meningitis, encephalitis, and brain infection. Severe *Listeria* infection is associated with a 20-30% mortality rate [9]. While previous case reports implicate colonoscopy in allowing the invasion of the bacteria across the intestinal epithelium, this patient’s blood cultures upon admission were positive, prior to the colonoscopy procedure [10]. Furthermore, Inoue et al. suggest that* L. monocytogenes* infection leads to worsening of UC flares [3]. It is possible that the *L. monocytogenes *infection triggered the UC flare in this patient and the increased inflammation allowed for the seeding of the bacteria in the bloodstream.

The duration of treatment for Listeriosis does not have specific guidelines due to the rarity of systemic *L. monocytogenes *infection. Treatment length should be tailored to the specific antibiotic regimen and severity of the patient’s clinical picture. One author suggests that for an immunocompetent patient with listeriosis, treatment should last 14 days. For patients who have weakened immune systems, treatment can range anywhere from three to six weeks [11]. A different cohort study suggested that the average treatment with amoxicillin was 16 days. However, for combination therapy with ampicillin and gentamicin, the average duration dropped to four days. In that study, 43% of listeriosis patients were under some type of immunosuppression [12]. Our patient has been receiving intravenous antibiotics, including ampicillin and gentamicin, for four days and was switched to high-dose amoxicillin for 14 days with plans for close follow-up, to ensure complete resolution of his infection.

## Conclusions

UC is a chronic inflammatory bowel disease characterized by bloody diarrhea and a relapsing/remitting chronic infection. *L. monocytogenes *is one of the most common foodborne infections in the United States, with a high mortality rate for systemic infections. This case report supports the findings that UC treatment with immune-modulating therapies can place patients at an increased risk for opportunistic infections, such as *L. monocytogenes, *and that *L. monocytogenes* infection can be associated with a UC flare.
